# Short birth spacing and its impact on maternal and child health in India with urban-rural variation: An epidemiological study using the National Family Health Survey Data

**DOI:** 10.1371/journal.pone.0325461

**Published:** 2025-06-27

**Authors:** Ramendra Nath Kundu, Juri Borah, Arna Chatterjee, Koel Mukherjee, Susmita Bharati, Md. Golam Hossain, Premananda Bharati

**Affiliations:** 1 Department of Anthropology, West Bengal State University, Berunanpukuria, West Bengal, India; 2 Department of Anthropology, Dr. Harisingh Gour Vishwavidyalaya (A Central University), Sagar, Madhya Pradesh, India; 3 Department of Anthropology, Gurucharan College, Silchar, Assam, India; 4 Anthropological Survey of India, SRC, Mysuru, India; 5 Anthropological Survey of India, North East Regional Center, Shillong, Meghalaya, India; 6 Sociological Research Unit, Indian Statistical Institute, Kolkata, West Bengal, India; 7 Health Research Group, Department of Statistics, University of Rajshahi, Rajshahi, Bangladesh; 8 Biological Anthropology Unit, Indian Statistical Institute, Kolkata, West Bengal, India; International Institute of Health Management Research - New Delhi, INDIA

## Abstract

**Background:**

Maintaining an appropriate gap between childbirths is essential for promoting the health of both the mother and the child. This practice lowers the risk of adverse maternal and child health outcomes. Inadequate birth spacing is a multifaceted issue linked to increase infant mortality and has the potential to impact the health and demographic landscape of the nation. This study aims to assess the prevalence of short birth spacing (SBS) in association with demographic and socio-economic factors in India with special reference to maternal and child health.

**Methods:**

This study investigated SBS in India using a cross-sectional design and data from the National Family Health Survey (NFHS-5). The analysis included data from 636,699 households, encompassing 724,115 women and 139,660 birth intervals. The primary outcome was SBS, and secondary outcomes included maternal undernutrition, full antenatal care (ANC), low birth weight (LBW), infant mortality, and child mortality. The analysis incorporated demographic and socio-economic variables as explanatory factors for SBS. Data analysis was conducted after checking its normality. The data characteristics were summarised and analysed using descriptive and inferential statistics.

**Results:**

In India, SBS is more prevalent (50.8%) with a median of 32 months. Bihar and Andhra Pradesh have shortest spacing (27 months), while Lakshadweep has longest (63.9 months). The Central zone has highest SBS prevalence (53.8%), especially in rural areas (55.9%). SBS is significantly associated with lack of maternal education, younger maternal age, unwanted pregnancies, larger families, and poor wealth index. Additionally, it is found that the explanatory variables have a significant impact on SBS, with area under the curve (AUC) covering 68% to 71% (p < 0.001). Furthermore, SBS has a detrimental effect on maternal and child health as secondary outcomes.

**Conclusion:**

This study emphasizes the substantial influence of socio-demographic factors on the practice of SBS. It is crucial to provide educational programs for mothers that focus on the importance of birth spacing and using family planning services. The study highlights the need to implement economic development initiatives within families to address the factors contributing to inadequate birth spacing. These measures enhance maternal and child health and align with the SDGs in India.

## Introduction

Maintaining optimal birth intervals is a significant health-promoting and potentially life-saving measure for mothers and children [1–3]. Optimal birth interval has been associated with reduced risk of maternal and child mortality as well as improved outcomes for both [4]. Hence, knowledge and awareness regarding maintaining optimum birth intervals are of utmost importance, as lengthening the birth interval depends upon the choice of couples. Understanding the factors influencing birth spacing is essential for developing effective public policies since they often appear to vary quite substantially across populations [5]. Several studies have identified key determinants of birth intervals, including the mother’s age at first birth, parity, previous birth interval, duration of breastfeeding, non-use of contraceptives mother’s working status, gender composition of the living children, place of residence, and exposure to mass media [6–9]. Previous research has shown that birth spacing is an independent risk factor for infant and child mortality [10,11], maternal health [12], preterm birth [13,14], congenital anomalies [15,16], and low birth weight [17–19]. The implications of closely spaced births go beyond health; they increase population growth, decrease economic development and women’s productivity, and increase the demand for natural resources [20].

According to the World Health Organisation (WHO) technical consultation report on birth spacing (i.e., birth-to-pregnancy interval), a minimum of 24 months is recommended for a healthy pregnancy. This indicates a birth-to-birth interval of 33 months, assuming a nine-month gestation period [21]. Further, the research conducted by USAID on optimal birth spacing suggested that waiting 3–5 years as the optimal birth spacing offers additional health benefits to the child [22]. This aligns with the findings of other research studies, demonstrating that birth intervals of 3–5 years are safer for both mother and infant than ≤2 years [23,24].

A significant disparity was observed in a comparative analysis of birth spacing practices between developing and developed countries [7]. The study revealed that women in developing countries like Egypt often prefer longer intervals between births than they experience in reality [25]. From a cursory glance at previous studies, it is observed that many mothers in different parts of the world are found to give birth to their babies within less than 2 years of gap [6,7,12,17,26]. This indicates the presence of influential factors shaping birth spacing decisions, potentially resulting in outcomes diverging from the mother’s intrinsic desires. In India, there is a campaign by the National Health Mission under governmental schemes that promotes 3 years of gap between births, highlighting the importance of birth spacing. Hence, to evaluate the current situation and design effectively, it is important to estimate the incidence of birth spacing in India and identify the factors that influence it. Additionally, it is crucial to investigate the effects of short birth spacing on child and maternal health.

The primary objective of this study is to assess the prevalence of short birth spacing (SBS) and its association with demographic and socio-economic factors in urban and rural areas of India using NFHS-5 data. Additionally, the study seeks to examine the impact of SBS on the health of mothers and the mortality of children.

## Methods

### Conceptual framework

**Fig 1:**
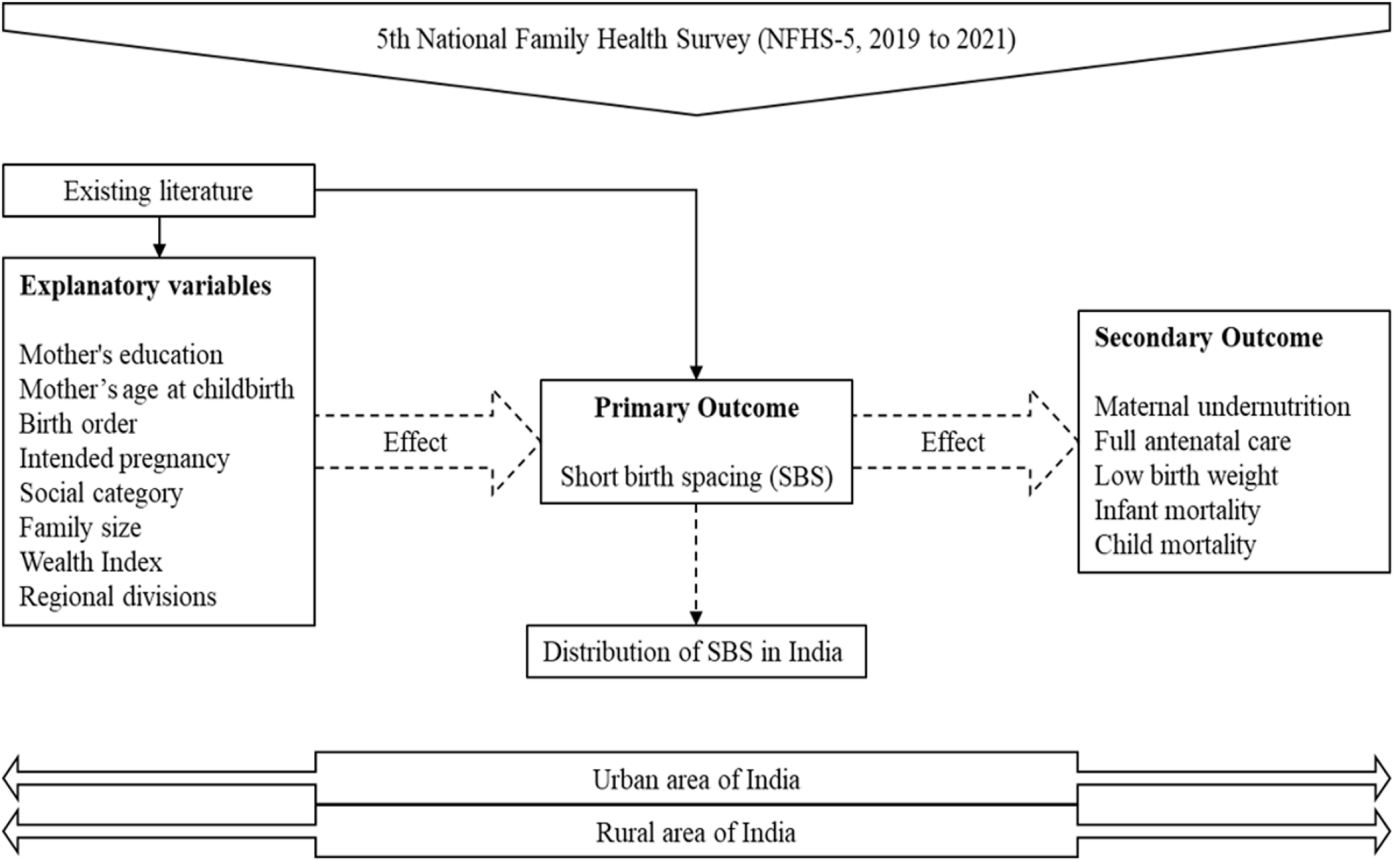
Conceptual framework.

The study aims to investigate the prevalence of SBS in both urban and rural areas of India using a cross-sectional approach (Fig 1). The association of demographic and socio-economic characteristics with SBS was developed based on existing literature, while maternal and child health association with SBS was developed independently. The study was based on data from the fifth National Family Health Survey conducted between 2019 and 2021 (NFHS 5). The latest distribution of SBS in India was established using NFHS 5 data, and the study also identified the demographic and socio-economic factors influencing SBS (Fig 1). This information is important for policymakers in developing strategies to address the issue of SBS in India.

### Study design and subject

The study employed a cross-sectional research design and obtained data from the National Family Health Survey of 2019−21 (NFHS-5). The survey was conducted by the International Institute for Population Sciences (IIPS). This comprehensive nationwide household survey utilized standardized questionnaires, sampling techniques, and field methodology in accordance with the guiding principle of the Demographic and Health Surveys (DHS) Program [27]. Initially, primary sampling units (PSUs) were selected based on Census data. Subsequently, households were randomly selected within these PSUs. The survey ensured appropriate representation by categorizing the population into strata based on factors such as rural/urban residence and state/region [27].

**Fig 2:**
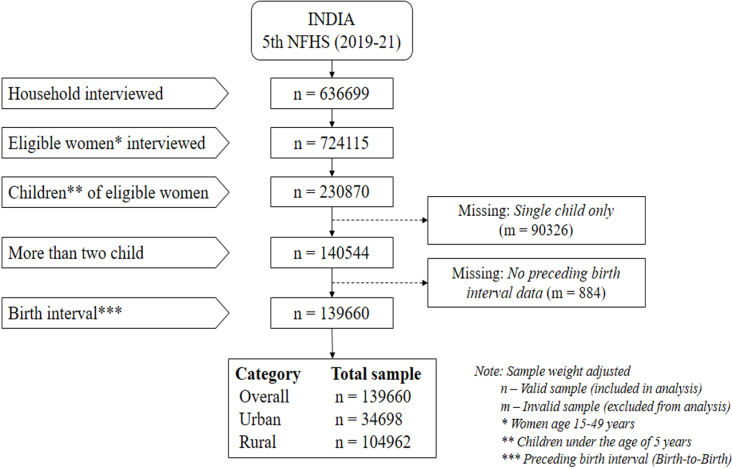
Procedure of the sample selection for the study.

Data was collected from 636,699 households, including 724,115 women [27]. Among the eligible women (aged 15–49 years), 230,870 children under the age of five were initially identified. Of these, 90,326 were excluded from the analysis as they were single children, and 884 were excluded due to the absence of preceding birth interval data. Ultimately, 139,660 children were included in the analysis, as in the flow chart of Fig 2.

### Unit-level variables

#### Outcome variable.

The study primarily focused on SBS, and it was the primary outcome variable, which refers to the interval of less than 33 months between the last child and their immediate elder sibling [28,29]. According to WHO reports, the interval between birth and conception should be at least 24 months in two consecutive births [21].

Additionally, the study examined secondary outcome variables such as maternal undernutrition, full antenatal care (ANC), low birth weight (LBW), infant mortality, and child mortality. Maternal undernutrition was assessed using body mass index (BMI), calculated by dividing weight in kilograms by height in meters squared (kg/m²). According to the WHO, a BMI value of less than 18.5 was considered underweight, indicating maternal undernutrition, while a value of 18.5 or higher was considered non-underweight [30]. A birth weight of less than 2500 grams was classified as low birth weight (LBW), while a birth weight of 2500 grams or more was considered normal [31,32]. Full antenatal care was described as having four or more antenatal visits, receiving at least one tetanus toxoid (TT) injection, and consuming iron folic acid (IFA) tablets or syrup for at least 100 days [27,33]. Infant mortality refers to the death between birth and the first birthday (0 to <12 months), while child mortality refers to the death of a child between their first and fifth birthday (1 to <5 years) [27,34,35].

In this study, the outcome variables were organized into primary and secondary groups based on the use of two-tiered binary logistic regression approach. The initial analysis focused on SBS as the primary outcome, investigating various exposures as explanatory variables to assess their effects. In the following analysis, we shifted our focus to maternal and child health as the secondary outcome, with SBS serving as the explanatory variable. The secondary outcome variables were subsequently affected by the primary outcome variable.

#### Grouping variable.

The study was conducted in India and involved categorizing participants based on their place of residence. The participants were grouped into urban and rural settings, with these categories serving as the grouping variables for the study. This approach enabled us to explore potential differences in birth spacing and health outcomes between these two settings.

#### Explanatory variables.

The study incorporated demographic and socio-economic variables as explanatory factors, drawing on existing research and data from the NFHS-5 dataset. These factors were explicitly categorized, and their definitions were upheld by leveraging or adapting from previous research. The factors have included mother’s education (no formal education, formal education), mother’s age at childbirth (<18, 18–25, > 25 years), birth order (≤2, ≥ 3), intended pregnancy (wanted, unwanted), social category (ST, SC, OBC, General), family size (≤4, > 4 members), wealth index (poor, middle, rich), regional divisions (central, east, north, northeast, south, west).

In this research, maternal education pertains to the formal academic achievements of the mother. No formal education refers to mothers who have never attended school, whereas formal education includes those who have completed formal schooling, such as primary, secondary, and higher. Maternal age was determined in relation to the birth of her child. When examining birth order, the first child was excluded, and the study focused on second and subsequent children, as a minimum of two children was necessary to analyze birth spacing. In the case of an intended pregnancy, if a woman indicated that her recent birth was the one she planned at the time, it was classified as a wanted birth. On the other hand, if the birth occurred before intended or was not intended at all, it was considered an unwanted birth [27]. The social category, as outlined in the Indian Constitution, was determined by whether the head of the household self-identified as a member of Scheduled Tribe (ST), Scheduled Caste (SC), or Other Backward Class (OBC). Households not falling under these categories were commonly considered as general or unreserved categories [36,37]. In the context of household size, families of four or fewer members were considered small, while those with more than four members were categorized as large families. The wealth index assesses a household’s prosperity by considering its assets, the quantity and variety of consumer goods, and housing characteristics, such as access to clean drinking water, toilet facilities, and quality of flooring. The scores were divided into five equal groups, with the most affluent families receiving the highest score (rich) and the least affluent receiving the lowest score (poor) [27]. The regions of India were classified according to the NFHS report [27].

### Statistical analysis

This study employed various statistical techniques to examine the relationship between SBS and explanatory variables, as well as secondary outcomes. The normality of the primary outcome variable was assessed using skewness, kurtosis, and Kolmogorov-Smirnov (K-S) statistics. Data were considered normal if the z values of skewness and kurtosis fell within ±1.96, and if the K-S test showed p > 0.05 [38]. Descriptive statistics, such as frequency, percentage, mean, standard deviation (SD), median, and interquartile range (IQR), were used to present the characteristics of the primary outcome variable. This study utilized non-parametric inferential statistics due to unmet parametric test assumptions. The proportional-Z method was employed to analyse the variations in percentages of different explanatory variables between SBS and non-SBS. This was based on the principle of the null hypothesis, H0: p_1_ - p_2_ = 0, where p_1_ represents the proportion from the first population and p_2_ represents the proportion from the second population. The selection of explanatory variables followed a thorough multicollinearity test using variance inflation factors (VIFs) and tolerance indices (TIs). Only variables with a VIF of less than 5.0 or a TI of greater than 0.20 were taken into consideration [39,40].

The study employed binary logistic regression analysis to explore the association between dependent and independent variables. This involved calculating both crude and adjusted odds ratios. The crude odds ratio (COR) was used to assess the individual effect of the independent variable, while the adjusted odds ratio (AOR) considered the cumulative effect of the independent variables [41,42]. Statistical significance was determined using a 95% confidence interval (CI), with a significance level of p ≤ 0.05. The model’s accuracy in multivariate analysis was evaluated through the sensitivity and specificity of the predicted probability values for the explanatory variables as illustrated in the receiver operating characteristic (ROC) curve. In order to ensure the precision of the findings, the sampling weight was adjusted during statistical analyses, and any missing values were excluded using list-wise exclusion. The statistical analyses were performed using STATA (version 17).

### Ethics approval and consent to participate

The present study relies on nationally representative data from the National Family Health Survey 2019–2021 (NFHS-5) obtained from the Demographic and Health Survey (DHS) and available in the public domain. The ICF Institutional Review Board (IRB) examined and approved the study design and participant confidentiality, the NFHS-5 data were already ethically approved; therefore use of these data no longer required any other ethical approval.

## Results

**Fig 3:**
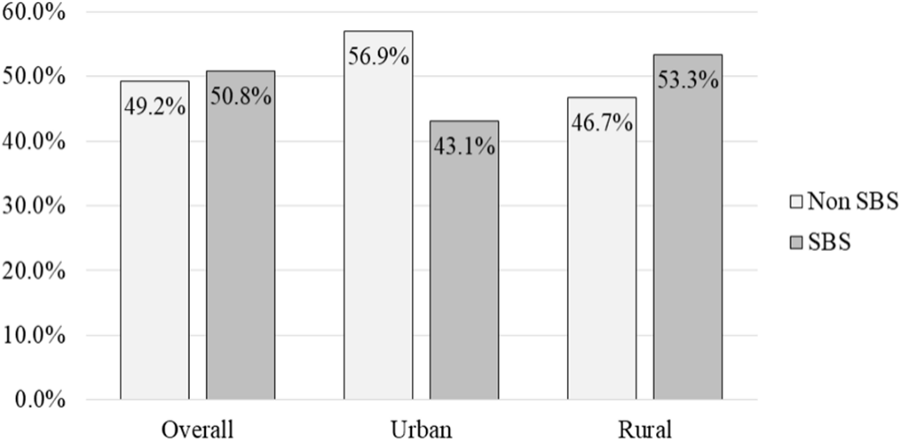
Distribution and urban-rural comparison of birth spacing in India.

The distribution of birth spacing in India has been presented in Table 1 and Fig 3. The prevalence of SBS in India was higher (50.8%) than that of non-SBS (49.2%). The SBS was found to be more commonly practiced in rural India (53.3%) than in urban India (43.1%) (Fig 3). The average birth spacing in India was 38.97 (CI: 38.84–39.09) months, with urban areas having 43.87 (CI: 43.58–44.16) months and rural areas having 37.35 (CI: 37.21–37.48) months (S1 Table).

**Table 1:** Birth spacing and its distribution in India as primary outcome.

Indian Population	Non-SBS N (%)	SBS N (%)
Overall	68746 (49.2)	70914 (50.8)
Urban	19752 (56.9)	14946 (43.1)
Rural	48994 (46.7)	55968 (53.3)

**Fig 4:**
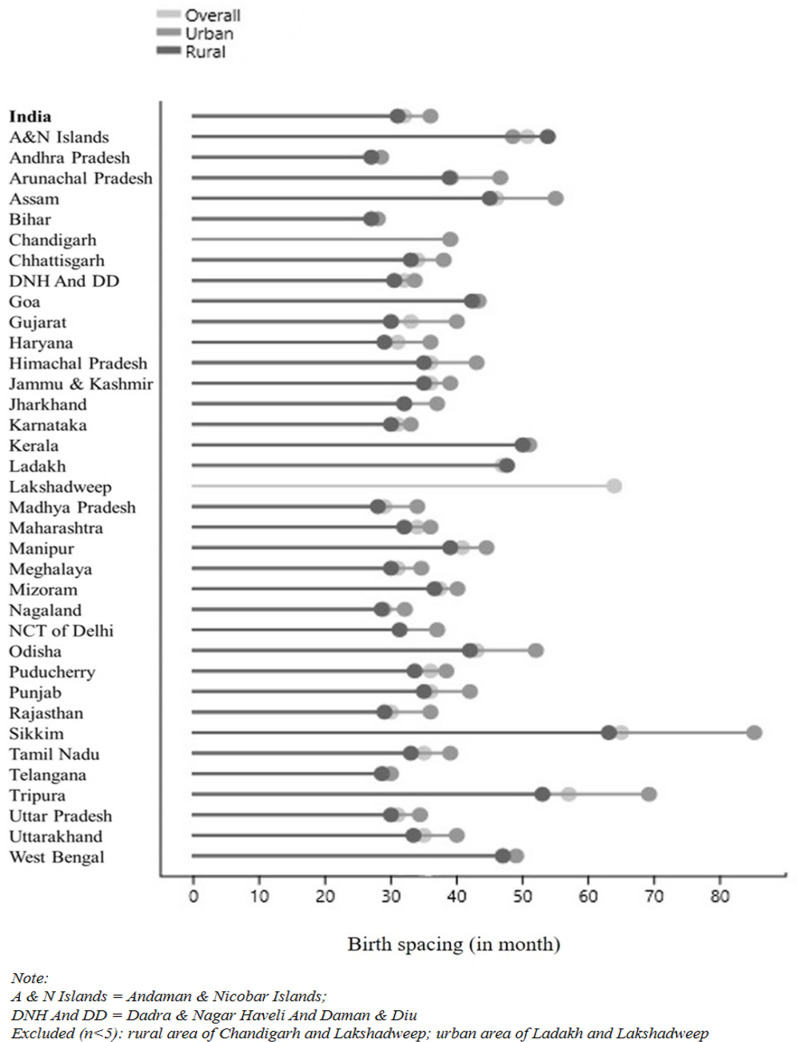
Distribution of median-birth spacing across the States/UTs.

The distribution of birth spacing across the States and Union Territories in Fig 4 considered median values. Since the data were not normally distributed, as indicated by Z-skewness and Z-kurtosis, along with the Kolmogorov-Smirnov (K-S) test showing significance (p < 0.001) in both urban and rural areas of India (S1 Table). States/UTs with a sample size of less than 5 were excluded from the figure, as mentioned in S2 Table.

The Fig 4 shows that the median birth spacing in India was 32 months overall, with 36 months in urban areas and 31 months in rural areas. The shortest birth spacing was observed in Bihar and Andhra Pradesh (27 months for both States), while the longest was found in Sikkim (65 months) and Lakshadweep (63.9 months). Additionally, Bihar and Andhra Pradesh had the shortest birth spacing in urban areas (28 months and 28.5 months) and rural areas (27 months for both States).

The distribution of birth spacing with various explanatory variables (including demographic and socio-economic characteristics) and secondary outcomes (such as maternal undernutrition and ANC, LBW, and mortality) were presented in Table 2.

**Table 2:** Distribution of studied variables according to SBS by overall, urban, and rural India.

Variable characteristics	Overall		Urban		Rural	
	SBS N (%)	Proportional-Z (p-value)	SBS N (%)	Proportional-Z (p-value)	SBS N (%)	Proportional-Z (p-value)
***Explanatory variables***						
Mother’s education						
No formal education	21359 (54.7)	18.50 (<0.001)	2840 (52.1)	3.10 (0.002)	18519 (55.1)	18.60 (<0.001)
Formal education	49556 (49.3)	−4.44 (<0.001)	12106 (41.4)	−28.99 (<0.001)	37449 (52.5)	13.34 (<0.001)
Mother’s age at childbirth						
<18 years	669 (88.4)	16.77 (<0.001)	115 (97.5)	7.51 (<0.001)	554 (86.7)	14.96 (<0.001)
18-25 years	39859 (66.9)	78.14 (<0.001)	8041 (64.5)	31.11 (<0.001)	31818 (67.6)	72.07 (<0.001)
>25 years	30386 (38.3)	−64.18 (<0.001)	6790 (30.7)	−53.55 (<0.001)	23596 (41.2)	−41.47 (<0.001)
Birth order						
≤ 2	37874 (49.4)	−3.32 (0.001)	9143 (41.7)	−24.26 (<0.001)	28731 (52.5)	11.69 (<0.001)
≥ 3	33040 (52.5)	12.52 (<0.001)	5803 (45.5)	−10.12 (<0.001)	27237 (54.3)	19.20 (<0.001)
Intended pregnancy						
Wanted	61245 (49.3)	−4.93 (<0.001)	12783 (41.3)	−30.17 (<0.001)	48462 (52.0)	12.20 (<0.001)
Unwanted	9669 (62.4)	29.94 (<0.001)	2163 (58.1)	9.76 (<0.001)	7506 (63.8)	28.84 (<0.001)
Social category						
ST	7650 (52.8)	6.72 (<0.001)	697 (48.9)	−0.83 (0.407)	6953 (53.3)	7.52 (<0.001)
SC	18226 (54.4)	16.05 (<0.001)	3480 (48.9)	−1.86 (0.063)	14747 (55.9)	19.04 (<0.001)
OBC	31734 (52.0)	9.88 (<0.001)	6568 (43.0)	−17.14 (<0.001)	25166 (55.0)	21.29 (<0.001)
General	10382 (44.6)	−16.39 (<0.001)	3363 (38.5)	−20.95 (<0.001)	7018 (48.2)	−4.34 (<0.001)
Family size						
≤ 4 members	12897 (43.4)	−22.56 (<0.001)	3204 (36.8)	−23.83 (<0.001)	9693 (46.2)	−10.98 (<0.001)
> 4 members	58017 (52.8)	18.54 (<0.001)	11742 (45.2)	−15.40 (<0.001)	46275 (55.1)	29.40 (<0.001)
Wealth Index						
Poor	39832 (55.4)	28.79 (<0.001)	3182 (55.8)	8.70 (<0.001)	36650 (55.4)	27.63 (<0.001)
Middle	13862 (52.2)	7.16 (<0.001)	3286 (49.0)	−1.64 (0.101)	10576 (53.4)	9.55 (<0.001)
Rich	17220 (41.8)	−32.85 (<0.001)	8478 (38.0)	−34.85 (<0.001)	8742 (46.2)	−10.43 (<0.001)
Regional divisions						
Central	22487 (53.8)	15.50 (<0.001)	3785 (45.4)	−8.37 (<0.001)	18703 (55.9)	21.44 (<0.001)
East	19418 (51.7)	6.59 (<0.001)	2521 (43.7)	−9.49 (<0.001)	16897 (53.1)	11.04 (<0.001)
North	9214 (50.5)	1.35 (0.177)	2336 (42.2)	−11.47 (<0.001)	6878 (54.2)	9.43 (<0.001)
Northeast	1583 (32.4)	−23.21 (<0.001)	171 (26.8)	−10.63 (<0.001)	1411 (33.2)	−20.76 (<0.001)
South	10319 (49.6)	−1.15 (0.25)	3478 (43.7)	−11.15 (<0.001)	6841 (53.2)	7.24 (<0.001)
West	7894 (48.4)	−4.09 (<0.001)	2655 (41.1)	−14.08 (<0.001)	5238 (53.1)	6.14 (<0.001)
***Secondary Outcome***						
Maternal undernutrition						
No	53844 (49.3)	−4.63 (<0.001)	12185 (42.1)	−26.56 (<0.001)	41659 (51.9)	10.75 (<0.001)
Yes	14879 (57.5)	23.86 (<0.001)	2020 (53.0)	3.70 (<0.001)	12859 (58.3)	24.32 (<0.001)
Full antenatal care						
No	41015 (49.7)	−1.72 (0.085)	8122 (43.3)	−18.18 (<0.001)	32892 (51.6)	8.07 (<0.001)
Yes	12877 (40.6)	−32.92 (<0.001)	3862 (34.0)	−32.48 (<0.001)	9015 (44.2)	−16.46 (<0.001)
Low birth weight						
No	50101 (49.2)	−5.11 (<0.001)	11124 (41.7)	−26.75 (<0.001)	38977 (51.8)	9.86 (<0.001)
Yes	11015 (51.3)	3.81 (<0.001)	2334 (43.6)	−9.29 (<0.001)	8681 (53.8)	9.63 (<0.001)
Infant mortality						
No	68055 (50.4)	2.94 (0.003)	14479 (42.8)	−26.22 (<0.001)	53576 (52.9)	18.42 (<0.001)
Yes	2860 (62.0)	15.85 (<0.001)	467 (54.3)	2.51 (0.012)	2392 (63.8)	16.30 (<0.001)
Child mortality						
No	67708 (50.3)	2.20 (0.028)	14422 (42.8)	−26.17 (<0.001)	53286 (52.9)	18.38 (<0.001)
Yes	346 (63.4)	6.05 (<0.001)	56 (49.6)	−0.09 (0.928)	290 (67.0)	6.70 (<0.001)

Note: *Null Hypothesis (H0): p1* *−* *p2* *=* *0, where p1 is the proportion of SBS and p2 is the proportion of Non-SBS.*

*[Proportion of p1 = %SBS/ 100, and Proportion of p2 = 1 – p1]*

The study found that the prevalence of SBS was significantly higher among mothers with no formal education, rural areas showed comparatively higher prevalence (55.1%) than in urban areas (52.1%). Younger mothers during the age at childbirth, especially those below 18 years old, were found to be the most vulnerable age group in India, with 88.4% prevalence of SBS, including 97.5% in urban areas and 86.7% in rural areas. On the other hand, mothers older than 25 years showed an improvement in both urban and rural areas, with a significant difference (proportional-Z, p < 0.001). The prevalence of SBS was higher after the second child (52.5%) compared to the first two children (49.4%) in India, including urban and rural areas. In the case of intended pregnancy, the unwanted pregnancy had a higher prevalence of SBS compared to the wanted pregnancy for both urban (58.1%) and rural (63.8%) areas. Among social categories, SC have the highest national exposure to SBS at 54.4%, followed by ST at 52.8% and OBC at 52.0%. The SBS was more common in large families (>4 members) compared to small families (≤4 members), with rural areas showing a higher prevalence (55.1%) than urban areas (45.2%). In poor and middle-class families, SBS was more prevalent nationally (55.4% and 52.2%), while in urban areas, only poor families showed higher prevalence. SBS was more common in the Central zone of India (53.8%), including urban (45.4%) and rural (55.9%) areas, followed by the Eastern zone (51.7%). The Northeast zone has a comparatively lower prevalence at 32.4%, including rural (33.2%) and urban (26.8%) areas.

As an exposure SBS affects maternal and child health variables, including mother’s undernutrition (BMI < 18.5 kg/m^2^), antenatal care, low birth weight, infant mortality, and child mortality. In India maternal undernutrition was more prevalent in SBS (57.5%), including urban (53.0%) and rural (58.3%) areas. In India, 49.7% of mothers who engaged in SBS did not attend the full ANC. This prevalence was higher in rural areas (51.6%) compared to urban areas (43.3%). Low birth weight children were more common in SBS, particularly in rural areas (53.8%), with a national prevalence of 51.3%. Infant mortality (IM) and child mortality (CM) were both more frequent in SBS, with the highest prevalence in rural areas (IM = 63.8%, CM = 67.0%) compared to urban areas (IM = 54.3%, CM = 49.6%).

### How demographic and socio-economic factors affect SBS

Table 3 illustrates the individual impact of explanatory variables on SBS in India, comparing urban and rural differences based on COR. It was observed that the mother’s education level was a common factor influencing SBS throughout India. The likelihood of SBS was 1.24 times higher (CI: 1.21–1.27) for non-educated mothers compared to those with educated mothers. This effect was more pronounced in urban areas (COR 1.54, CI: 1.45–1.63) than in rural areas (COR 1.11, CI: 1.08–1.14). Among younger mothers, those under 18 years during childbirth were 3.74 times (CI: 3.00–4.68) more likely to practice SBS, with significantly higher odds in urban areas by 20.22 times (CI: 6.57–62.27) compared to rural areas (COR 3.12, CI: 2.48–3.93). Conversely, mothers above 25 years were 69% less likely to have SBS in India (COR 0.31, CI: 0.30–0.31), including urban and rural areas.

**Table 3:** Crude odds ratio of the explanatory variables on SBS in overall India with comparing urban and rural variations.

Explanatory variables	Overall		Urban		Rural	
	COR (95% CI)	p-value	COR (95% CI)	p-value	COR (95% CI)	p-value
Mother’s education (Formal education ®)						
No formal education	1.24 (1.21, 1.27)	<0.001	1.54 (1.45, 1.63)	<0.001	1.11 (1.08, 1.14)	<0.001
Mother’s age at childbirth (18–25 years ®)						
<18 years	3.74 (3.00, 4.68)	<0.001	20.22 (6.57, 62.27)	<0.001	3.12 (2.48, 3.93)	<0.001
>25 years	0.31 (0.30, 0.31)	<0.001	0.24 (0.23, 0.26)	<0.001	0.34 (0.33, 0.35)	<0.001
Birth order (≥ 3 ®)						
≤ 2	0.88 (0.86, 0.90)	<0.001	0.86 (0.82, 0.89)	<0.001	0.93 (0.91, 0.95)	<0.001
Intended pregnancy (Wanted ®)						
Unwanted	1.71 (1.65, 1.77)	<0.001	1.97 (1.84, 2.11)	<0.001	1.63 (1.57, 1.70)	<0.001
Social category (General ®)						
ST	1.39 (1.34, 1.45)	<0.001	1.53 (1.36, 1.71)	<0.001	1.22 (1.17, 1.28)	<0.001
SC	1.48 (1.43, 1.53)	<0.001	1.53 (1.44, 1.63)	<0.001	1.36 (1.31, 1.42)	<0.001
OBC	1.35 (1.31, 1.39)	<0.001	1.20 (1.14, 1.27)	<0.001	1.31 (1.26, 1.36)	<0.001
Family size (≤ 4 members ®)						
> 4 members	1.46 (1.42, 1.50)	<0.001	1.42 (1.35, 1.49)	<0.001	1.43 (1.39, 1.48)	<0.001
Wealth index (Rich ®)						
Poor	1.73 (1.69, 1.77)	<0.001	2.06 (1.94, 2.18)	<0.001	1.44 (1.40, 1.49)	<0.001
Middle	1.52 (1.48, 1.57)	<0.001	1.56 (1.48, 1.65)	<0.001	1.33 (1.28, 1.39)	<0.001
Regional divisions (South ®)						
Central	1.18 (1.14, 1.22)	<0.001	1.07 (1.00, 1.14)	0.035	1.11 (1.07, 1.16)	<0.001
East	1.09 (1.05, 1.13)	<0.001	1.00 (0.93, 1.07)	0.984	1.00 (0.96, 1.04)	0.920
North	1.04 (1.00, 1.08)	0.059	0.94 (0.88, 1.01)	0.075	1.04 (0.99, 1.09)	0.115
Northeast	0.49 (0.46, 0.52)	<0.001	0.47 (0.39, 0.57)	<0.001	0.44 (0.41, 0.47)	<0.001
West	0.95 (0.92, 0.99)	0.024	0.90 (0.84, 0.96)	0.002	1.00 (0.95, 1.05)	0.943

*Note: Null Hypothesis (H0):* $COR\;=\left(\frac{odds\;p1,\;\;for\;primary\;outcome}{odds\;p2,\;\;for\;primary\;outcome}\right)=1$

*Where, p1 is the odds of each tested explanatory variable’s category, p2 is the odds of corresponding reference category, and primary outcome variable is SBS.*

*COR = Crude Odds Ratio, which indicates individual association; 95% CI = 95% Confidence interval; ® = Reference category*

The likelihood of SBS during the first two childbirths was 12% lower (COR 0.88, CI: 0.86–0.90) nationwide, with a similar pattern observed in urban and rural areas. SBS was more prevalent in instances of unwanted pregnancy, with 1.71 times higher odds (CI: 1.65–1.77) nationwide and even higher in urban areas (COR 1.97, CI: 1.84–2.11). In comparison to the General caste community within the social categories in India, the SC community was 1.48 times more likely (CI: 1.43–1.53) to practice SBS, followed by ST (COR 1.39, CI: 1.34–1.45) and OBC (COR 1.35, CI: 1.31–1.39) communities. In the urban areas, the prevalence was notably equal among the ST and SC communities. In large families (>4 members) the likelihood of practicing SBS was 1.46 times higher (CI: 1.42–1.50) compared to smaller families (≤4 members), and this pattern was consistent in both urban and rural areas.

The wealth index also played a significant role in SBS; in India, poor families were 1.73 times more likely to practice SBS than wealthier families, and this likelihood increased to over two times in urban poor households (COR 2.06, CI: 1.94–2.18). This pattern was also evident among middle-class families, indicating the need for targeted interventions based on socio-economic status. Regarding the regional prevalence of SBS in India, the Northeast region exhibited a lower prevalence (COR 0.49, CI: 0.46–0.52) of SBS compared to the South region, followed by the West (COR 0.95, CI: 0.92–0.99). In contrast, the Central (COR 1.18, CI: 1.14–1.22) and East (COR 1.09, CI: 1.05–1.13) regions demonstrated higher rates of SBS practice. This highlights the need for region-specific interventions to address the varying prevalence rates.

Table 4 presents the adjusted model, demonstrating the influence of explanatory variables on SBS across India and comparing urban and rural differences based on AOR. After adjusting the explanatory variables, the following association of SBS was found. Non-educated mothers showed a higher likelihood of practicing SBS, with a 1.10 times higher prevalence in India (CI: 1.07–1.14), with urban areas showed comparatively higher odds (AOR 1.23, CI: 1.14–1.31) than rural areas (AOR 1.08, CI: 1.04–1.11). Younger mothers were identified as more susceptible to practicing SBS, with a prevalence that was four times higher in India (AOR 4.11, CI: 3.23–5.24), 17.54 times higher in urban areas (CI: 5.67–54.27), and 3.58 times higher in rural areas (CI: 2.79–4.60). In contrast, mothers aged above 25 years were found to be 74% less likely to practice SBS nationally (AOR 0.26, CI: 0.25–0.27); similarly, the likelihood was 77% lower in urban areas (AOR 0.23, CI: 0.22–0.24) and 73% lower in rural areas (AOR 0.27, CI: 0.27–0.28).

**Table 4:** Adjusted odds ratio of the explanatory variables on SBS in overall India with comparing urban and rural variations.

Explanatory variables	Overall		Urban		Rural	
	AOR (95% CI)	p-value	AOR (95% CI)	p-value	AOR (95% CI)	p-value
Mother’s education (Formal education ®)						
No formal education	1.10 (1.07, 1.14)	<0.001	1.23 (1.14, 1.31)	<0.001	1.08 (1.04, 1.11)	<0.001
Mother’s age at childbirth (18–25 years ®)						
<18 years	4.11 (3.23, 5.24)	<0.001	17.54 (5.67, 54.27)	<0.001	3.58 (2.79, 4.60)	<0.001
>25 years	0.26 (0.25, 0.27)	<0.001	0.23 (0.22, 0.24)	<0.001	0.27 (0.27, 0.28)	<0.001
Birth order (≥ 3 ®)						
≤ 2	0.70 (0.68, 0.72)	<0.001	0.79 (0.74, 0.83)	<0.001	0.68 (0.66, 0.70)	<0.001
Intended pregnancy (Wanted ®)						
Unwanted	1.65 (1.59, 1.71)	<0.001	1.88 (1.74, 2.03)	<0.001	1.59 (1.52, 1.66)	<0.001
Social category (General ®)						
ST	1.16 (1.11, 1.22)	<0.001	1.32 (1.16, 1.50)	<0.001	1.14 (1.08, 1.20)	<0.001
SC	1.19 (1.15, 1.24)	<0.001	1.19 (1.11, 1.28)	<0.001	1.19 (1.14, 1.24)	<0.001
OBC	1.17 (1.14, 1.21)	<0.001	1.08 (1.01, 1.14)	0.017	1.19 (1.14, 1.24)	<0.001
Family size (≤ 4 members ®)						
> 4 members	1.39 (1.35, 1.43)	<0.001	1.39 (1.31, 1.47)	<0.001	1.38 (1.33, 1.43)	<0.001
Wealth index (Rich ®)						
Poor	1.50 (1.46, 1.55)	<0.001	1.67 (1.55, 1.79)	<0.001	1.35 (1.30, 1.41)	<0.001
Middle	1.31 (1.26, 1.35)	<0.001	1.30 (1.22, 1.38)	<0.001	1.22 (1.16, 1.27)	<0.001
Regional divisions (South ®)						
Central	0.97 (0.93, 1.00)	0.078	0.92 (0.86, 0.98)	0.016	0.99 (0.95, 1.04)	0.707
East	0.76 (0.73, 0.79)	<0.001	0.66 (0.60, 0.71)	<0.001	0.80 (0.76, 0.83)	<0.001
North	0.98 (0.94, 1.03)	0.446	0.83 (0.77, 0.90)	<0.001	1.04 (0.98, 1.09)	0.197
Northeast	0.42 (0.38, 0.46)	<0.001	0.40 (0.32, 0.50)	<0.001	0.43 (0.39, 0.47)	<0.001
West	0.86 (0.82, 0.90)	<0.001	0.78 (0.72, 0.84)	<0.001	0.92 (0.86, 0.97)	0.003

*Note: Null Hypothesis (H0):* $AOR\;=\left(\frac{adjusted\;odds\;p1,\;\;for\;primary\;outcome}{adjusted\;odds\;p2,\;\;for\;primary\;outcome}\right)=1$

*Where, p1 is the adjusted odds of each tested explanatory variable’s category, p2 is the adjusted odds of corresponding reference category, and primary outcome variable is SBS.*

*AOR = Adjusted Odds Ratio, which indicates cumulative association; 95% CI = 95% Confidence interval; ® = Reference category*

Nationally, the likelihood of SBS occurring in the first two childbirths was found to be 30% lower (AOR 0.70, CI: 0.68–0.72), with a similar pattern observed in both urban and rural areas. The intended pregnancy found as a significant factor; SBS was more prevalent in unwanted pregnancy, being 1.65 times more likely in India (CI: 1.59–1.71), including urban and rural areas. Among the social categories in India, SC was 1.19 times (CI: 1.15–1.24), and OBC was 1.17 times (CI: 1.14–1.21) more likely to practice SBS. The likelihood of SBS was 1.39 times (CI: 1.35–1.43) higher in large families compared to small families.

The study also revealed that poor and middle-class families in India were 1.50 times (CI: 1.46–1.55) and 1.31 times (CI: 1.26–1.35) more likely to practice SBS than wealthier families. Furthermore, the vulnerability of poor families was higher in urban areas (AOR 1.67, CI: 1.55–1.79) than in rural areas (AOR 1.35, CI: 1.30–1.41). However, the Northeast region in India was 58% less prevalent to SBS (AOR 0.42, CI: 0.38–0.46) than the South, followed by the East at 24% (AOR 0.76, CI: 0.73–0.79) and the West at 14% (AOR 0.86, CI: 0.82–0.90).

### Area under the ROC curve

**Fig 5:**
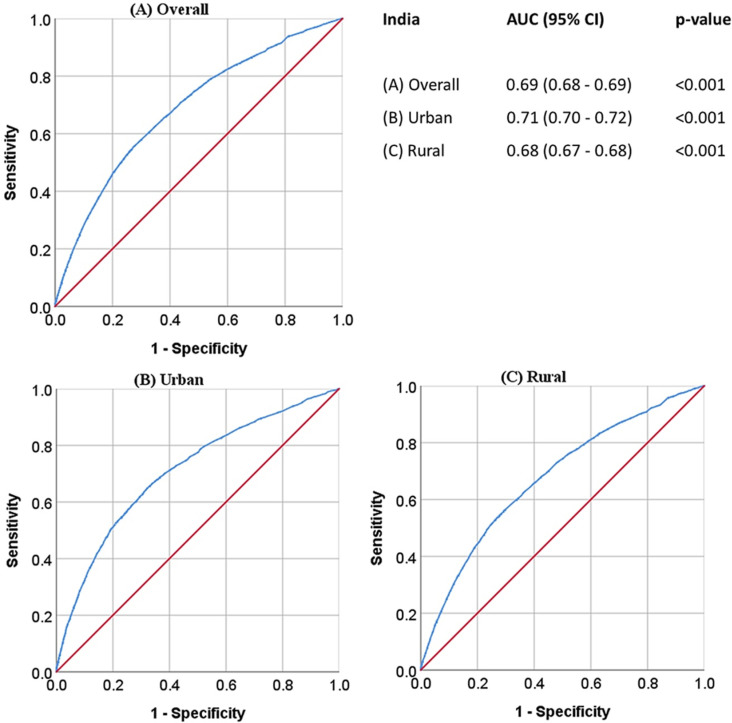
AUC for the predicted probability of explanatory variables (adjusted) on SBS.

The impact of the predicted probability of explanatory variables on SBS is illustrated in Fig 5 through the ROC curve. The predicted probability levels demonstrate the cumulative effect of all explanatory variables on the outcome variable. It was found that the explanatory variables had a significant impact on SBS, with the AUC being statistically significant (p < 0.001). The AUC of predicted probability for the explanatory variables in each curve exceeded 50%. The analysis revealed that the maximum impact observed in urban areas was 71%, while in rural areas it was 68%. On a national level, the impact was determined to be 69%.

### SBS affects maternal and child health

**Fig 6:**
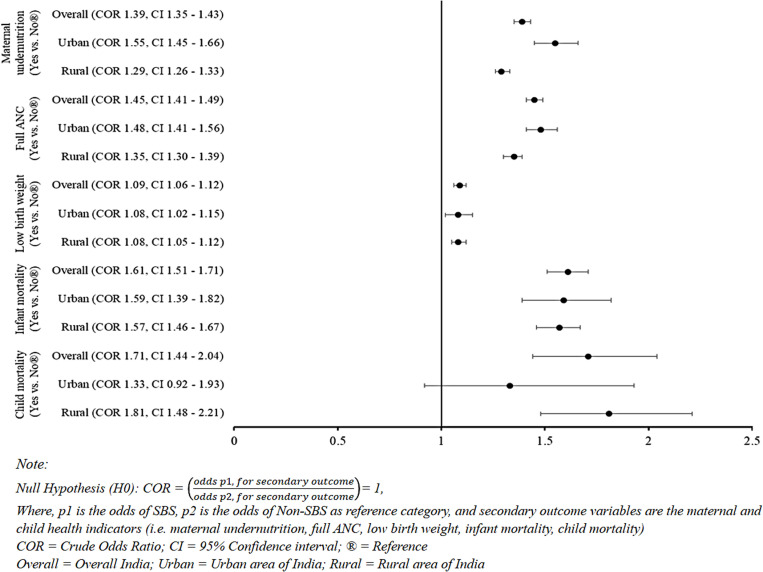
Effect of SBS on maternal and child health in India with urban and rural variations.

The impact of SBS on maternal and child health is presented in Fig 6 and the S3 Table. These findings illustrate how the practice of SBS influences maternal undernutrition and antenatal care (ANC), low birth weight, and infant and child mortality. The data indicates that practicing SBS increased the likelihood of maternal undernutrition, nationally being 1.39 times (CI: 1.35–1.43) more prevalent than in non-SBS cases. This prevalence was notably higher in urban areas (COR 1.55, CI: 1.45–1.66) compared to rural areas (COR 1.29, CI: 1.26–1.33). The practice of SBS increased the likelihood of hindering mothers from receiving full ANC, nationally 1.45 times more prevalent (CI: 1.41–1.49), which was higher in urban (COR 1.48, CI: 1.41–1.56) than in rural (COR 1.35, CI: 1.30–1.39) areas. Children born with SBS were 1.09 times (CI: 1.06–1.12) more likely to have low birth weight, regardless of whether they were born in urban or rural areas. However, SBS also increases the likelihood of infant and child mortality. In India, infant mortality was 1.61 times (CI: 1.51–1.71) more likely, and child mortality was 1.71 times (CI: 1.44–2.04) more likely among those born with SBS. Infant mortality was more prevalent in urban areas (COR 1.59, CI: 1.39–1.82), while child mortality was more prevalent in rural areas (COR 1.81, CI: 1.48–2.21) (Fig 6).

## Discussion

This study examined the prevalence of birth spacing and its determinants in India and explored the impact of SBS on maternal and child health. The finding indicates over half of the total births in India (50.8%) occurred within short intervals. This result was consistent with research conducted in Uganda, where the prevalence of SBS was reported as 52.4% [6]. In India, SBS was found to be most prevalent in the Central zone (53.8%), closely by the Eastern zone (51.7%). In terms of social categories in India, ST community was more vulnerable, had the highest national exposure to SBS (54.4%), and followed by SC (52.8%) and OBC (52.0%).

The prevalence of SBS was found to be higher in rural areas of India (53.3%) compared to urban areas (43.1%). Similar findings were observed in other low- and middle-income countries (LMICs) such as Ethiopia (rural: 58.2%, urban: 41.1%), Tanzania (rural: 50.0%, urban: 45.0%), Bangladesh (rural: 27.0%, urban: 24.0%), and Nigeria (rural: 20.7%, urban: 20.3%) [7,43–45]. This pattern was consistent with observations in Sub-Saharan Africa [46,47].

The median birth spacing in India was found to be 32 months overall, with urban areas showing a slightly longer median of 36 months compared to rural areas at 31 months. There were notable variations across different states, with Bihar and Andhra Pradesh exhibiting the shortest birth spacing at 27 months. Additionally, similar median birth spacing patterns were observed in other studies from Tanzania (33.4 months), and East Ethiopia (31 months) [43,48].

The study findings indicated that mothers who gave birth at a younger age and had no formal education were leading to them being more prone to practicing SBS. These findings align with research from other LMICs such as Jordan, Saudi Arabia, Northern Iran, Tanzania, Nepal, Bangladesh, Ghana, Southern Ethiopia, Eastern Ethiopia, Nigeria, and Mozambique, which further reinforces the validity of present findings [3,22,24,26,43,45,48–52]. After the birth of a second child, there is a higher observed prevalence of SBS compared to the birth of the first two children. This finding corroborated with research conducted in Tanzania and Ghana, which also indicated that women who gave birth to higher-order babies were more likely to have SBS [43,52].

The results of our study suggested that women who experience unplanned pregnancies were more likely to have SBS than those who plan their pregnancies. This observation may be attributed to the propensity of women who plan their pregnancies to adhere to child spacing recommendations and utilize contraceptive methods to achieve their desired family planning goals. The present study supported by similar studies conducted in Ghana [53]. Furthermore, our study indicates that there was a higher likelihood of SBS in larger families (more than 4 members) when compared to smaller families. It was found that low and middle-class families in India were more inclined to practice SBS compared to wealthier families. This attributed to the fact that women from wealthier families tend to have significantly longer birth intervals due to factors such as education and lifestyle. The findings of the present study were consistent with those of prior research conducted across Saudi Arabia [50], Bangladesh [26], Ethiopia [54], Nigeria [45], Northwest Ethiopia [55], and Ghana [52].

The results indicated that the impact of SBS on maternal undernutrition is a major concern. Closely spaced pregnancies can compromise maternal health by limiting the time for nutritional recovery. This depletion can negatively impact the mother’s well-being and the health of her developing baby [56]. A study conducted in Ethiopia also reported maternal nutritional deficiencies associated with SBS [57]. Furthermore, it was found that children born with SBS were more likely to have low birth weight. This increased risk stems from inadequate maternal weight gain during such pregnancies. The mother’s body may not have sufficient time to replenish essential nutrients, hindering fetal development and potentially leading to low birth weight [58]. In previous studies conducted in various countries, including Sudan [19], Tanzania [59], and Bangladesh [18], similar findings were reported. These studies revealed that SBS can hinder mothers from accessing full antenatal care (ANC). However, it has been noted that infants and children born with SBS in India were more prone to higher mortality rates. A significant relationship between SBS and infant mortality has also been documented in other countries, such as Bangladesh [18], Ethiopia [10], Pakistan [60], and Ghana [52].

### Strengths and limitations

This study presents nationally representative data on SBS in India, providing comprehensive insights that align with global patterns. However, the research is not without its limitations, which is the key for further investigation. First, the study relied on secondary data from the NFHS survey and could be influenced by recall bias. Second, the study’s cross-sectional design limits the ability to establish causal relationships between short birth intervals and health outcomes. The analysis faced challenges due to multicollinearity issue and missing data, which affected the inclusion of several factors, such as contraceptive method use, breastfeeding practices, child survival outcomes, and age at marriage. In addition, further consideration of the scientific quality of the data from the NFHS, focusing on the trend of birth spacing, could improve our overall understanding of birth spacing in India.

## Conclusion

The findings of our study provide insights into the high prevalence of short birth intervals in India, revealing variations across rural and urban areas and different geographical regions. Our analysis has identified various socioeconomic and demographic factors that contribute to this phenomenon. These factors include non-literate or less educated mothers, young maternal age, belonging to socially disadvantaged groups, unintended pregnancies, poor economic status, high birth order, and larger family size, all of which appear to influence birth spacing patterns among women in India. The study has evidenced the significant and observable negative impacts of short birth intervals, with implications such as maternal nutritional deficiencies, low birth weight babies, incomplete antenatal care, and increased rates of infant and child mortality being apparent among the participants. Therefore, it is important for the healthcare system in India to focus on educating young men and women about the significance of optimal birth spacing and to provide comprehensive information on appropriate family planning services to improve maternal and child health in particular and the country in general. This intervention not only addresses the specific health concerns, but also contributes to India’s progress towards achieving four of the United Nations’ Sustainable Development Goals (SDGs): No poverty (SDG-1), Good health and well-being (SDG-3), Quality education (SDG-4), and Reduced inequalities (SDG-10).

## Supporting information

S1 Table Descriptive statistics of data normality for preceding birth spacing (in months). (DOCX)

S2 Table Distribution of median birth spacing (in months) according to States/UTs in India. (DOCX)

S3 Table Effect of short birth spacing on maternal and child health as secondary outcome. (DOCX)

## Abbreviations

A & N Islands: Andaman & Nicobar Islands
ANC: antenatal care
AOR: adjusted odds ratio
AUC: area under the curve
BMI: body mass index
CI: confidence interval
CM: child mortality
COR: crude odds ratio
DHS: Demographic and Health Surveys
DNH and DD: Dadra & Nagar Haveli And Daman & Diu
IFA: iron folic acid
IIPS: International Institute for Population Sciences
IM: infant mortality
IQR: interquartile range
K-S: Kolmogorov-Smirnov
LBW: low birth weight
LMIC: low- and middle-income countries
NFHS: National Family Health Survey
NFHS-5: National Family Health Survey, between 2019 and 2021
OBC: other backward class
PSU: primary sampling unit
ROC curve: receiver operating characteristic curve
SBS: short birth spacing
SC: scheduled caste
SD: standard deviation
SDGs: sustainable development goals
ST: scheduled tribe
TI: tolerance indices
TT: tetanus toxoid
USAID: United States Agency for International Development
UT: union territory
VIF: variance inflation factors
WHO: World Health Organization
