# Supplementary material for: Short birth spacing and its impact on maternal and child health in India with urban-rural variation: An epidemiological study using the National Family Health Survey Data
Source: PLoS One. 2025 Jun 27;20(6):e0325461. doi: 10.1371/journal.pone.0325461 (PMC12204571; doi:10.1371/journal.pone.0325461)
Supplement: S1 Table — (DOCX) [file pone.0325461.s001.docx]

| **S1 Table:** Descriptive statistics of data normality for preceding birth spacing (in months) | | | | | | |
| --- | --- | --- | --- | --- | --- | --- |
| Characteristics |  | Overall |  | Urban |  | Rural |
| Sample size |  | 139660 |  | 34698 |  | 104962 |
| Mean (95% CI) |  | 38.97 (38.84, 39.09) |  | 43.87 (43.58, 44.16) |  | 37.35 (37.21, 37.48) |
| Median (IQR) |  | 32.00 (25.00) |  | 36.00 (32.00) |  | 31.00 (24.00) |
| Skewness (SE) |  | 1.99 (0.007) |  | 1.71 (0.013) |  | 2.08 (0.008) |
| Z-Skewness |  | 284.29 |  | 131.54 |  | 260.00 |
| Kurtosis (SE) |  | 6.19 (0.013) |  | 4.37 (0.026) |  | 6.96 (0.015) |
| Z-Kurtosis |  | 476.15 |  | 168.08 |  | 464.00 |
| K-S (p-value) |  | 0.14 (<0.001) |  | 0.12 (<0.001) |  | 0.14 (<0.001) |
| *Note: CI = Confidence Interval; IQR = Interquartile Range; K-S = Kolmogorov-Smirnova, SE = Standard Error* | | | | | | |
