# Supplementary material for: Short birth spacing and its impact on maternal and child health in India with urban-rural variation: An epidemiological study using the National Family Health Survey Data
Source: PLoS One. 2025 Jun 27;20(6):e0325461. doi: 10.1371/journal.pone.0325461 (PMC12204571; doi:10.1371/journal.pone.0325461)
Supplement: S2 Table — (DOCX) [file pone.0325461.s002.docx]

S2 **Table:** Distribution of median birth spacing (in months) according to States/UTs in India

| State/union territory |  | Overall | |  | Urban | |  | Rural | |
| --- | --- | --- | --- | --- | --- | --- | --- | --- | --- |
|  |  | N | Median |  | N | Median |  | N | Median |
| **India** |  | 139660 | 32.0 |  | 34698 | 36.0 |  | 104962 | 31 |
| A & N Islands |  | 20 | 50.7 |  | 10 | 48.5 |  | 10 | 53.8 |
| Andhra Pradesh |  | 4069 | 27.0 |  | 1110 | 28.5 |  | 2959 | 27.0 |
| Arunachal Pradesh |  | 104 | 39.2 |  | 13 | 46.6 |  | 91 | 38.9 |
| Assam |  | 3292 | 46.0 |  | 306 | 55.0 |  | 2986 | 45.0 |
| Bihar |  | 21325 | 27.0 |  | 2678 | 28.0 |  | 18647 | 27.0 |
| Chandigarh |  | 88 | 39.0 |  | 86 | 39.0 |  | 2 | 83.0 |
| Chhattisgarh |  | 3085 | 34.0 |  | 558 | 38.0 |  | 2527 | 33.0 |
| DNH And DD |  | 53 | 32.0 |  | 24 | 33.6 |  | 29 | 30.5 |
| Goa |  | 87 | 43.0 |  | 55 | 43.3 |  | 32 | 42.3 |
| Gujarat |  | 5686 | 33.0 |  | 1954 | 40.0 |  | 3732 | 30.0 |
| Haryana |  | 2646 | 31.0 |  | 734 | 36.0 |  | 1912 | 29.0 |
| Himachal Pradesh |  | 502 | 36.0 |  | 58 | 43.0 |  | 444 | 35.0 |
| Jammu & Kashmir |  | 922 | 36.0 |  | 182 | 39.0 |  | 740 | 35.0 |
| Jharkhand |  | 4415 | 32.0 |  | 657 | 37.0 |  | 3758 | 32.0 |
| Karnataka |  | 5696 | 31.0 |  | 2054 | 33.0 |  | 3642 | 30.0 |
| Kerala |  | 2542 | 50.0 |  | 1221 | 51.0 |  | 1321 | 50.0 |
| Ladakh |  | 16 | 46.9 |  | 3 | 59.2 |  | 13 | 47.6 |
| Lakshadweep |  | 5 | 63.9 |  | 4 | 69.9 |  | 1 | 127.0 |
| Madhya Pradesh |  | 8491 | 29.0 |  | 1785 | 34.0 |  | 6706 | 28.0 |
| Maharashtra |  | 10491 | 34.0 |  | 4424 | 36.0 |  | 6067 | 32.0 |
| Manipur |  | 278 | 40.8 |  | 81 | 44.5 |  | 197 | 39.0 |
| Meghalaya |  | 646 | 31.0 |  | 82 | 34.6 |  | 564 | 30.0 |
| Mizoram |  | 122 | 37.4 |  | 60 | 40.1 |  | 62 | 36.6 |
| Nagaland |  | 133 | 29.0 |  | 31 | 32.1 |  | 102 | 28.6 |
| NCT of Delhi |  | 1730 | 37.0 |  | 1678 | 37.0 |  | 52 | 31.3 |
| Odisha |  | 4059 | 43.0 |  | 549 | 52.0 |  | 3510 | 42.0 |
| Puducherry |  | 74 | 36.0 |  | 51 | 38.4 |  | 23 | 33.6 |
| Punjab |  | 2247 | 36.0 |  | 806 | 42.0 |  | 1441 | 35.0 |
| Rajasthan |  | 8984 | 30.0 |  | 1640 | 36.0 |  | 7344 | 29.0 |
| Sikkim |  | 26 | 65.0 |  | 7 | 85.2 |  | 19 | 63.1 |
| Tamil Nadu |  | 5462 | 35.0 |  | 2325 | 39.0 |  | 3137 | 33.0 |
| Telangana |  | 2951 | 29.0 |  | 1180 | 30.0 |  | 1771 | 28.6 |
| Tripura |  | 285 | 57.0 |  | 57 | 69.2 |  | 228 | 53.0 |
| Uttar Pradesh |  | 30248 | 31.0 |  | 6000 | 34.4 |  | 24248 | 30.0 |
| Uttarakhand |  | 1102 | 35.0 |  | 350 | 40.0 |  | 752 | 33.4 |
| West Bengal |  | 7778 | 47.0 |  | 1885 | 49.0 |  | 5893 | 47.0 |
| *Note: A & N Islands: Andaman & Nicobar Islands;*  *DNH And DD = Dadra & Nagar Haveli And Daman & Diu* | | | | | | | | | |
