# Supplementary material for: Short birth spacing and its impact on maternal and child health in India with urban-rural variation: An epidemiological study using the National Family Health Survey Data
Source: PLoS One. 2025 Jun 27;20(6):e0325461. doi: 10.1371/journal.pone.0325461 (PMC12204571; doi:10.1371/journal.pone.0325461)
Supplement: S3 Table — (DOCX) [file pone.0325461.s003.docx]

| **S3 Table:** Effect of short birth spacing on maternal and child health as secondary outcome | | | | | | | | | | |
| --- | --- | --- | --- | --- | --- | --- | --- | --- | --- | --- |
| Health and mortality  Characteristics | |  | Overall | |  | Urban | |  | Rural | |
|  |  |  | COR (95% CI) | p-value |  | COR (95% CI) | p-value |  | COR (95% CI) | p-value |
| Maternal undernutrition | |  |  |  |  |  |  |  |  |  |
|  | No |  | 1 |  |  | 1 |  |  | 1 |  |
|  | Yes |  | 1.39 (1.35, 1.43) | <0.001 |  | 1.55 (1.45, 1.66) | <0.001 |  | 1.29 (1.26, 1.33) | <0.001 |
| Full ANC | |  |  |  |  |  |  |  |  |  |
|  | Yes |  | 1 |  |  | 1 |  |  | 1 |  |
|  | No |  | 1.45 (1.41, 1.49) | <0.001 |  | 1.48 (1.41, 1.56) | <0.001 |  | 1.35 (1.30, 1.39) | <0.001 |
| Low birth weight | |  |  |  |  |  |  |  |  |  |
|  | No |  | 1 |  |  | 1 |  |  | 1 |  |
|  | Yes |  | 1.09 (1.06, 1.12) | <0.001 |  | 1.08 (1.02, 1.15) | 0.009 |  | 1.08 (1.05, 1.12) | <0.001 |
| Infant mortality | |  |  |  |  |  |  |  |  |  |
|  | No |  | 1 |  |  | 1 |  |  | 1 |  |
|  | Yes |  | 1.61 (1.51, 1.71) | <0.001 |  | 1.59 (1.39, 1.82) | <0.001 |  | 1.57 (1.46, 1.67) | <0.001 |
| Child mortality | |  |  |  |  |  |  |  |  |  |
|  | No |  | 1 |  |  | 1 |  |  | 1 |  |
|  | Yes |  | 1.71 (1.44, 2.04) | <0.001 |  | 1.33 (0.92, 1.93) | 0.127 |  | 1.81 (1.48, 2.21) | <0.001 |
| *Note: Null Hypothesis (H0): COR =* $\left( \frac{odds p1, for secondary outcome}{odds p2, for secondary outcome} \right)$*= 1,*  *Where, p1 is the odds of SBS, p2 is the odds of Non-SBS as reference category, and*  *secondary outcome variables are the maternal and child health indicators*  *(i.e. maternal undernutrition, full ANC, low birth weight, infant mortality, child mortality)* | | | | | | | | | | |
